# Cyclic Thrombocytopenia Misdiagnosed as Immune Thrombocytopenia (ITP): Diagnostic and Therapeutic Lessons

**DOI:** 10.7759/cureus.97667

**Published:** 2025-11-24

**Authors:** Sheilabi Seeburun, Eunseuk Lee, Sultan Salameh, Hussam Eltoukhy

**Affiliations:** 1 Internal Medicine, Rutgers Health Community Medical Center, Toms River, USA; 2 Internal Medicine, Rutgers - The State University of New Jersey, New Brunswick, USA; 3 Hematology and Oncology, Rutgers Cancer Institute of New Jersey, Newark, USA; 4 Hematology and Oncology, University Hospital - Rutgers New Jersey Medical School, Newark, USA

**Keywords:** avatrombopag, case report, cyclic thrombocytopenia, cyclosporine a, danazol, eltrombopag, hematology, immune thrombocytopenia, thrombopoietin receptor agonists

## Abstract

Cyclic thrombocytopenia (CTP) is a rare hematological disorder characterized by periodic fluctuations in platelet counts, resulting from diverse causes, including endocrine, immune-mediated, and idiopathic mechanisms. The cyclical nature of CTP often leads to misdiagnosis as immune thrombocytopenia (ITP), resulting in inappropriate long-term treatments and associated complications.

We report the case of a 60-year-old woman who experienced lifelong platelet variability, including during pregnancy and post-menopause, after being diagnosed with ITP in her early 20s. Over decades, she underwent extensive ITP-directed therapies, including corticosteroids, intravenous immunoglobulin (IVIG), rituximab, splenectomy, and thrombopoietin receptor agonists (TPO-RAs) like eltrombopag and avatrombopag. Persistent platelet cycling prompted a reevaluation of her condition, leading to a revised diagnosis of CTP. Her treatment was adjusted by increasing the avatrombopag dose, with additional consideration for cyclosporine A (CSA) and danazol if platelet cycling persisted. This case emphasizes the diagnostic challenges posed by CTP, which is often overlooked until ITP treatments fail. Regular biweekly platelet monitoring may facilitate earlier diagnosis. Better clinical awareness and clearer diagnostic guidelines are needed to reduce misdiagnosis and prevent unnecessary interventions.

## Introduction

Cyclic thrombocytopenia (CTP) is an exceedingly rare hematological disorder, with fewer than 70 reported cases in the literature [1]. It is defined by recurrent, periodic oscillations in platelet levels, with counts plummeting to <5 × 10^9^/L during nadirs and rapidly rebounding to normal or elevated levels within weeks. This fluctuation often leads to a misdiagnosis of primary immune thrombocytopenia (ITP). In a Canadian registry of 614 thrombocytopenia patients (<150 × 10^9^/L), 44% were diagnosed with ITP, while only 0.7% were identified as having CTP [2].

In this report, we present a case illustrating the clinical course, diagnostic pitfalls, and therapeutic considerations in CTP, emphasizing the importance of distinguishing it from ITP to prevent unnecessary immunosuppressive exposure.

## Case presentation

A 60-year-old Caucasian woman with a past medical history of attention-deficit/hyperactivity disorder, migraines, carpal tunnel syndrome, lumbar disc herniation, and osteopenia was initially diagnosed with ITP at the age of 19 after presenting with menorrhagia and petechiae. Initial laboratory evaluation revealed isolated thrombocytopenia with otherwise normal blood counts and peripheral smear findings. Bone marrow biopsy demonstrated normal cellularity and megakaryocytes, consistent with ITP. She was initially treated with prednisone and achieved an appropriate yet short-lived hematologic response. She was subsequently treated with danazol (an androgen derivative) for two years without sustained remission and underwent splenectomy in 1987, which resulted in a durable response that lasted until her first pregnancy in 1989.

During her first two pregnancies, she experienced recurrent thrombocytopenia with platelet counts ranging from 20 to 40 × 10^3^/µL without bleeding tendencies, and she was treated with corticosteroids, resulting in temporary stabilization. However, during her third pregnancy, her platelet counts dropped to 1-5 × 10^3^/µL, and she developed steroid resistance, prompting initiation of intravenous immunoglobulin (IVIG), administered as three-day courses every 10 days during crisis periods. Over time, she became refractory to IVIG as well and subsequently received a full course of rituximab (an anti-CD20 monoclonal antibody) in the mid-2000s, which was complicated by aseptic meningitis. Given the adverse event, further courses of rituximab were deferred. 

**Figure 1:**
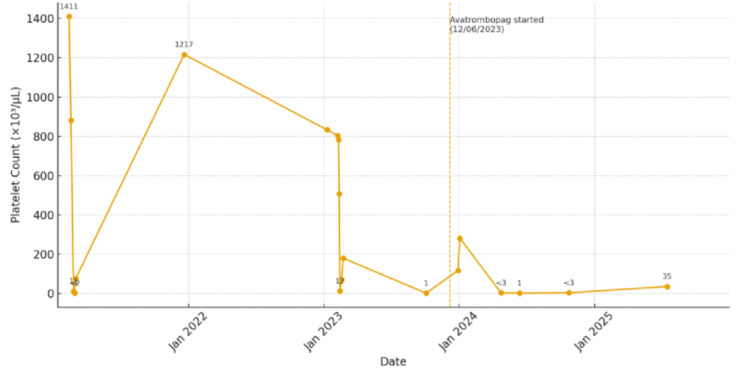
Fluctuations in Platelet Count Over Time Although much of her diagnostic history spans several decades, the most detailed laboratory data were available from February 2021 to December 2025, when more frequent monitoring captured the persistent platelet cycling shown in this figure. It graphically illustrates these oscillations over time, alternating severe thrombocytopenic nadirs (<10 × 10^3^/µL) and marked rebound thrombocytosis (>1000 × 10^3^/µL). The vertical dashed line indicates initiation of avatrombopag therapy on December 6, 2023.

In 2013, she was treated with eltrombopag (a thrombopoietin receptor agonist (TPO-RA)) at a dose alternating between 25 and 37.5 mg daily, after declining romiplostim (a TPO-RA). Despite treatment, her platelet levels continued to fluctuate markedly (Figure 1), cycling monthly from <10 × 10^3^/µL - associated with oral blood blisters, petechiae, and easy bruising - to peaks exceeding 1.3 × 10^6^/µL, associated with headaches. This pattern was initially believed to correlate with her menstrual cycles; however, the cyclical platelet variability persisted after menopause. She was also treated with aspirin 81 mg daily for thromboprophylaxis and tranexamic acid (an antifibrinolytic agent) 650 mg twice daily as needed for mucocutaneous bleeding, along with intermittent rescue treatment with dexamethasone for acute bleeding episodes. 

In 2023, eltrombopag was discontinued, and she was transitioned to another TPO-RA, avatrombopag 20 mg every other day, while continuing aspirin and tranexamic acid. Despite therapy, platelet cycling persisted, prompting re-evaluation and repeated workup, including HIV, hepatitis, thyroid function tests, thyroid peroxidase antibodies, autoimmune disorders such as systemic lupus erythematosus and rheumatoid arthritis, vitamin B12 level, folate level, a tick-borne panel, and flow cytometry analysis for acute leukemias and myeloid/lymphoid neoplasms, all of which were unremarkable. The patient denied alcohol consumption, antibiotic use, recent hospitalization with heparin injection, acute viral or bacterial illnesses, and recent travel to dengue-endemic areas. Her medications included multivitamins, amphetamine-dextroamphetamine, and a calcium supplement. Physical examination and CT scan of the abdomen did not reveal splenomegaly. Further workup revealed a positive T-cell receptor (TCR) gamma gene rearrangement by polymerase chain reaction. Her diagnosis was revised to CTP, and her avatrombopag dose was increased to 20 mg daily, with cyclosporine A (CSA), a calcineurin inhibitor immunosuppressant, and danazol considered as potential treatment options.

## Discussion

Clinical presentation 

CTP is typically diagnosed late in its course, often after standard ITP treatments fail [3]. Patients experience bleeding ranging from mild mucocutaneous episodes to severe hemorrhagic events during thrombocytopenic phases, with rebound thrombocytosis increasing the risk of thrombotic complications [4,5]. 

Pathophysiology

The pathogenesis of CTP remains incompletely understood and likely multifactorial [3]. Reported mechanisms include immune-mediated platelet destruction through immunoglobulin G (IgG) autoantibodies directed against platelet surface glycoproteins [6], bone marrow abnormalities such as megakaryocytic hypoplasia [7], and TCR rearrangements suggesting an immune-mediated cytotoxic process [1,3]. Some patients demonstrate an inverse correlation between platelet counts and TPO levels, indicating defective regulation of platelet production [1,3], while rare cases have shown mutations in the TPO receptor gene (MPL) [8], leading to impaired signaling and platelet homeostasis.

A subset of female patients experiences platelet cycling in synchrony with the menstrual cycle, typically peaking mid-cycle, although in some individuals the cyclic pattern persists after menopause, suggesting additional non-hormonal regulatory mechanisms [9]. Associations have also been described with autoimmune thyroid disease [1], hematologic malignancies such as T-cell large granular lymphocytic leukemia [1] and, rarely, cyclic neutropenia [10], underscoring the heterogeneity of this disorder.

Diagnosis

Kyrle and Eichinger [3] summarized the following clinical and laboratory features that should prompt clinicians to suspect CTP: inexplicable platelet fluctuations despite appropriate ITP therapy; persistent thrombocytosis while on TPO-RA treatment; a mild bleeding phenotype during severe thrombocytopenia; menstrual-synchronous fluctuations; TCR rearrangements; blood or thyroid gland disorders; fluctuations in white cell or reticulocyte counts; inverse cycling of platelet and TPO levels; and periodic megakaryocytic hypoplasia or aplasia.

Our patient exhibited several of these hallmark features. She demonstrated marked cyclic platelet oscillations despite receiving corticosteroids, IVIG, rituximab, splenectomy, and TPO-RAs, and while on eltrombopag, she continued to have extreme rebound thrombocytosis. Her bleeding phenotype often remained relatively mild, limited to mucocutaneous bleeding and menorrhagia when platelet counts dropped below 10 × 10^3^/µL. Additionally, all other causes of thrombocytopenia, blood disorders, and disorders of the thyroid gland were ruled out, and she was found to have a clonal TCR-gamma rearrangement. Although the cyclic pattern of her platelet counts was initially attributed to menstrual physiology, the persistence of oscillations after menopause strongly suggested a non-hormonal immune or marrow-regulatory process.

As outlined in published algorithms by Kyrle and Eichinger [3], once first- and second-line ITP therapies fail, clinicians should reassess the pattern of platelet fluctuation and can even consider temporarily withholding therapy while monitoring platelet counts at least weekly, preferably twice weekly, for several weeks [3].

Treatment 

Treatment strategies focus on managing the cyclical nature of platelet counts rather than following standard ITP protocols. In a large review by Go [11], none of the 26 patients demonstrated a sustained response to corticosteroids, and similar findings were reported by Kyrle and Eichinger [3], in which all institutional patients failed to respond to prednisolone. Likewise, splenectomy offers limited benefit, with only one of 19 patients in Go’s cohort showing a transient improvement [11]. In our case, the patient initially responded to corticosteroids and danazol, but relapsed shortly after splenectomy, consistent with the literature indicating poor long-term efficacy of these interventions.

TPO-RAs, such as romiplostim, eltrombopag, and avatrombopag, have been increasingly used in both ITP and CTP. Reports describe patients who failed to maintain stable platelet counts despite prolonged TPO-RA therapy, and some even developed thromboembolic events due to rebound thrombocytosis [3,12]. Since eltrombopag was ineffective in our case, the patient was transitioned to avatrombopag due to its favorable safety profile and lack of dietary restrictions [13]. Despite dose escalation to 20 mg daily, platelet cycling persisted, further supporting that TPO-RAs do not fully suppress cyclic variability.

Given these observations, alternative immunomodulatory agents, such as CSA and danazol, remain reasonable options for refractory CTP. CSA has shown partial efficacy in several reported cases, likely through suppression of clonal T-cell activity [14-16], while danazol has induced prolonged remissions of one to five years in isolated reports [17,18]. In our patient, sequential therapy with CSA is being considered to achieve sustained platelet stability and minimize the risks associated with extreme oscillations in case of persistent avatrombopag failure.

## Conclusions

Timely recognition of platelet cycling through routine biweekly monitoring can aid in earlier identification of CTP after failed ITP-directed therapies. Some patients respond to CSA or danazol, but in the majority of patients, platelet cycling continues. Future research should aim to uncover the molecular mechanisms underlying CTP, particularly the roles of cytokines and immune dysregulation, to develop therapies addressing its cyclical nature and improve outcomes for this challenging condition.
